# Autogenous Fiber Laser Welding of 316L Austenitic and 2304 Lean Duplex Stainless Steels

**DOI:** 10.3390/ma13132930

**Published:** 2020-06-30

**Authors:** Michał Landowski, Aleksandra Świerczyńska, Grzegorz Rogalski, Dariusz Fydrych

**Affiliations:** Faculty of Mechanical Engineering, Gdańsk University of Technology, 80-233 Gdańsk, Poland; aleksandra.swierczynska@pg.edu.pl (A.Ś.); grzegorz.rogalski@pg.edu.pl (G.R.); dariusz.fydrych@pg.edu.pl (D.F.)

**Keywords:** laser welding, austenitic stainless steel, lean duplex stainless steel, dissimilar welded joints, mechanical properties, microstructure

## Abstract

This study presents results of experimental tests on quality of dissimilar welded joints between 316L austenitic and 2304 lean duplex stainless steels, welded without ceramic backing. Fiber laser welded butt joints at a thickness of 8 mm were subjected to non-destructive testing (visual and penetrant), destructive testing (static tensile test, bending test, and microhardness measurements) and structure observations (macro- and microscopic examinations, SEM, element distribution characteristics, and ferrite content measurements). Non-destructive tests and metallographic examinations showed that the welded joints meet the acceptance criteria for B level in accordance with EN ISO 13919–1 standard. Also the results of the destructive tests confirmed the high quality of the joints: specimens were fractured in base material with lower strength—316L austenitic stainless steel and a 180° bending angle was obtained confirming the high plasticity of the joints. Microscopic examination, SEM and EDS analysis showed the distribution of alloying elements in joints. The microhardness of the autogenous weld metal was higher by about 20 HV0.2 than that of the lean duplex steel. Ferrite content in the root was about 37% higher than in the face of the weld. The Schaeffler phase diagram was used to predict the phase composition of the welded joints and sufficient compliance with the magnetic method was found. The presented procedure can be used for welding of 316L–2304 stainless steels dissimilar welded joints of 8 mm thickness without ceramic backing.

## 1. Introduction

Lasers as a heat source have been widely applied in industrial processes [1,2]. Currently, they are used for welding, cutting, remelting, and various processes with surface modification of materials [3,4,5,6]. Laser sources are characterized by: high energy density, high efficiency, large temperature gradients, high repeatability of the process, and formation of a narrow heat affected zone (HAZ). Many authors report beneficial changes in the properties of various materials subjected to laser treatment in such sectors as: medical, energy, ocean engineering, etc. [7,8,9]. The area of application of various technological laser solutions includes processing of many types of metals and their alloys, ceramics, polymers, and composites [1,5,10,11], including underwater local dry welding and wet welding [12,13]. Limiting the disadvantageous effects of technological use of lasers in some applications, e.g., the small width of weld, can be obtained by combining it with other heat sources to create hybrid processes. The most popular solutions include laser-metal active gas arc welding (MAG) and laser- tungsten inert gas arc welding (TIG) processes [14,15,16].

Often, high-alloy steels, especially stainless steels, are subjected to laser treatment. Stainless steels occupy an irreplaceable position in many branches of industry [17]. These steels are classified, among others according to the criteria of chemical composition and structure into: ferritic, martensitic, austenitic, and duplex (ferritic-austenitic) steels [18,19,20]. Austenitic steels are the most popular group of high-alloy stainless steels. The content of chromium (above 18%) and nickel (minimum 8%) provides the structure stability and the corrosion resistance [21,22,23]. In order to improve the properties of these steels, alloying elements: molybdenum, titanium, and niobium are introduced. The last two are excellent stabilizers and reduce the risk of intergranular corrosion. Austenitic steels are characterized by high corrosion resistance, good strength, and plastic parameters, hence their wide application. Improvement of mechanical properties can be achieved through the use of cold forming, and increase in corrosion resistance through the use of heat treatment. The group of austenitic stainless steels is considered well weldable by using many processes and maintaining an appropriate technological regime that includes all treatments related to the welding process, e.g., the prefabrication of the elements to be welded by using appropriate tools, the use of the consumables with an appropriate chemical composition, the control of the heat input and others. The main risks directly associated with the welding process are the formation of hot cracks and intergranular corrosion. General guidelines for welding this group of material can be found, among others, in many scientific reports [17,18,24], regulations and standards, e.g., EN 1011–3.

Duplex ferritic-austenitic steels are becoming increasingly popular due to their specific properties. They combine the advantages of other groups of high-alloy steels and the most important of them are high strength with good plasticity and corrosion resistance [25,26,27]. The two-phase structure is obtained by appropriate selection of the ratio of ferrite-forming elements (mainly Cr—21–28%) and austenite-forming elements (Ni—1.5–8%, N—0.05–0.3%). Depending on the content of these elements, duplex steels are divided into lean duplex, duplex, super duplex, and hyper duplex. Lean duplex steels represent a reasonable compromise between the need to provide the appropriate strength properties, desired corrosion resistance, and the requirement of low material costs [28]. Compared to austenitic stainless steels, they have higher strength parameters besides plasticity and higher resistance to stress corrosion. Steels from this group are characterized by good weldability, provided that the technological regime is maintained: the control of the value of heat input, the use of appropriate welding consumables dedicated to welded grade, the dilution rate, the suitable dimensions of the welding groove, the use of the appropriate type of shielding gas on the face and root side, maintaining high purity of welded components and other [29,30,31].

Duplex steels are sensitive to structural changes resulting from the welding thermal cycle. This can reduce the mechanical properties of the joints as well as their corrosion resistance. High cooling rate of duplex steel joints may result in higher ferrite content in HAZ and in the weld [31]. According to ASTM E562 standard, no more than 70% of ferrite is recommended, and according to Norskom M-601 it should be in the range of 30–70%.

Despite the fact that duplex steels are considered sensitive to high cooling rate, they can be successfully welded in conditions of large negative temperature gradients, e.g., under water and using concentrated heat sources [32,33,34]. In addition, a significant problem during welding is the risk of chromium nitride precipitation, as well as the occurrence of micro–areas depleted in Cr and Ni. Guidelines for welding duplex steels can be found in scientific reports [17,19], regulations and standards, e.g., EN 1011–3, Annex C.

In industrial applications (for example in: power generation, nuclear, petrochemical, aerospace, and shipbuilding sector) there is often a need to perform various variants of dissimilar joints [28,35,36]. A particular group of dissimilar joints are connections between steels from different groups of stainless steels. Of these, joints of the austenitic steel type with ferritic, martensitic and duplex steel grades are most often made. For joining stainless steels with other metals in addition to laser welding also other methods can be used: metal active gas arc welding (MAG), tungsten inert gas arc welding (TIG), shielded metal arc welding, arc stud welding, friction welding, friction stir welding, diffusion welding, explosive welding, and furnace brazing. For welding different grades of austenitic and duplex steels, many variants of technologies were used, which led to obtain joints with various morphology, mechanical properties, and corrosion resistance [37,38,39,40,41,42,43,44].

Welding of dissimilar stainless steel joints can be done between two main types of filler metals: austenitic (e.g., 309L) or duplex (e.g., 2209). Vincente et al. stated that the higher corrosion resistance of joints made by the MAG process is favored by the use of duplex steel consumable [45]. Similarly, Rahmani et al. recommend using duplex instead of austenitic consumable when using the TIG process [46]. However, among the various austenitic consumables, the most beneficial, both in terms of strength properties, as well as morphology and corrosion resistance, is the use of 309L [47,48] or super austenitic 904L filler metal [49]. The literature also describes attempts to weld such a material combination without filler material: using different variants of the TIG process [50,51] or concentrated welding sources [33,52,53]. Ridha Mohammed et al. stated that the mechanical properties of the duplex-austenitic steel fiber laser joints were better compared with the base materials (BM) because of the small HAZ [52]. In the same work, the authors found the presence in welded joints the regions with a completely austenitic solidification mode which were susceptible to solidification cracking. An important observation is that when laser welding is used for super duplex and austenitic steels, the ferrite/austenite phase balance is not significantly changed by different heat input values [39]. Existence of an unmixed zone that originated from each base material was stated by Chun et al. [53]. The approximately 50:50 ferrite/austenite microstructural balance can be reached with solution annealing in the range of 1050–1100 °C, and it is especially important in autogenous welds [33]. Saravanan et al. stated that the higher microhardness of the weld zone is attributed by the formation of finer and uniform grains following high cooling rate [54].

Although researches on laser welding of dissimilar stainless steels have been reported, the study on structure and properties of laser beam welded austenitic/lean duplex steel joints is still relatively rarely mentioned. Therefore, the present work aims to show the ability of making 316L austenitic stainless steel–2304 lean duplex stainless steel dissimilar joints using the autogenous fiber laser welding process.

## 2. Materials and Methods 

The test pieces were a flat plates with thickness of 8 mm made of 316L austenitic stainless steel (1.4404, UNS S31603) and 2304 lean duplex stainless steel (1.4362, UNS S32304) in delivery condition. Both materials were after heat treatment: 316L steel after solution annealing from 1050 °C and 2304 steel was hot rolled and solution annealed. The chemical composition of the used materials in accordance with the inspection certificate (spectral analysis) and the requirements of the EN 10088-2:2014 standard (minimum and maximum wt. %) are given in Table 1. The mechanical properties of the materials in accordance with the requirements of the EN 10088–2–2014 standard are presented in Table 2. Chromium and nickel equivalents (Creq and Nieq) were calculated according to [55].

**Figure 1:**
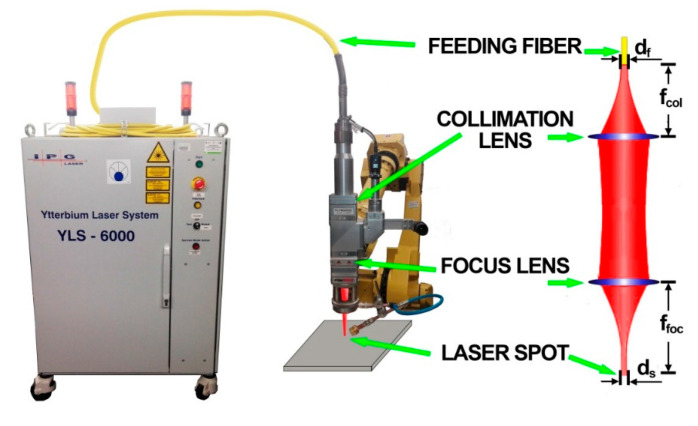
Experimental set-up for fiber laser welding [56].

A high power continuous wave ytterbium fiber laser IPG Photonics YLS–6000 (IPG Photonic, Oxford, MS, USA) with a maximum power of 6 kW and a wavelength of 1070 nm was used (Figure 1). The joints were made at 6 kW laser output power. The laser beam was delivered through the optical feeding fiber (transmitting fiber) of 300 µm core diameter. A welding head having a focal length of 250 mm and a focus collimator lens 150 mm was used. Focusing position was set on the surface of the plate. Welding speed was set to 25 mm/s (1.5 m/min). Welding process was autogenous—without filler metal. Shielding gas was supplied by a gas nozzle mounted at laser head to avoid the oxidation of weld beads. The flow rate of shielding gas—argon 5.0 (I1 in accordance with ISO 14175)—was set at 16 L/min. Test pieces were welded without using a ceramic backing or forming gas.

In order to identify and then eliminate any troubles with welding process the first stage of research consisted of making preliminary test joints. Initial joints were made to determine the values of significant variables, such as focal length, laser power and welding speed. The parameters of laser welding for austenitic stainless steel and lean duplex stainless steel differ, therefore, after initial experiments with similar materials, parameters matching both grades were selected. Before welding, the elements were cleaned with abrasive paper and degreased with acetone. Preliminary tests on stainless steels showed the presence of spatter if the surface was not cleaned properly just before welding.

To assess the quality of the welded joints, non-destructive tests (NDT) were performed: visual testing (VT)—according to the EN ISO 17637 standard and penetrant testing (PT)—according to the EN ISO 571–1 standard. Tensile and bending tests were conducted at ambient temperature of 20 °C using Instron 1195 (INSTRON, Norwood, MA, USA) universal testing machine in accordance with EN ISO 6892–1 and EN ISO 4136 standards. For bending test according to EN ISO 15614–11 bend former with a diameter of ϕ = 27 mm was used (the bend former diameter for materials with elongation above 25% is four times the specimen thickness). The acceptance criterion was defined as a bend angle α = 180°. Specimens in the face and root areas were slightly grinded to remove notches before performing tensile and three-point bending tests.

In order to prevent changes in the material structure under the influence of temperature during cutting, the specimens for metallographic examinations (according to EN ISO 17639) were cut mechanically in a direction transverse to the welding axis at the cutting machine with intensive cooling. Then it was grinded on abrasive papers with gradation 600–2400 and polishing on a polishing cloth using an aqueous suspension of 3 µm diamonds. This preparation of the specimen was followed by two-stage etching. The first stage was etching to reveal the austenite grain boundaries for which a mixture of 50 mL of boiling water, 3 mL of HNO_3_, and 1 mL of HF was used. The etching was performed by a two-minute immersion of specimens in an 80 °C temperature solution. Then the specimen was thoroughly rinsed and cooled under water. The next stage was etching in Beraha-type reagent (85 mL of water, 15 mL of HCl, and 1 g K_2_S_2_O_5_). Etching was carried out by about 1-3 min immersion in 20 °C temperature solution (until the corresponding colored structure was obtained on the specimen). At the end the specimen was thoroughly rinsed and dried with a stream of compressed air. Macroscopic metallographic observations were performed using DSLR Nikon d7000 with Tamron 90 mm f/2.8 macro lens (Nikon Corporation, Tokyo, Japan), while metallographic microscopy tests were done on a light microscope (LM) Olympus BX51 (Olympus, Tokyo, Japan). It offers imaging in a bright field, dark field and polarized light. The tests were also carried out using the scanning electron microscope JOEL JSM-7800F (SEM) with the EDAX adapter (Japan Electronics Corporation, Tokyo, Japan) enabling EDS analysis.

Microhardness measurements—HV0.2 were carried out using FM-800 tester with a load of F = 1.9614 N (Future-Tech, Tokyo, Japan). 

The ferrite number was determined using a Fischer Feritscope FMP30 for both base materials and weld (Helmut Fischer GmbH Institut für Elektronik und Messtechnik 71069 Sindelfingen, Germany). The Feritscope was calibrated on calibration standards prior to measurements. The ferrite content measurements were carried out in accordance with ISO 17655, at 6 measuring points for each place of measure.

## 3. Results and Discussion

### 3.1. Non-Destructive Tests 

**Figure 2:**
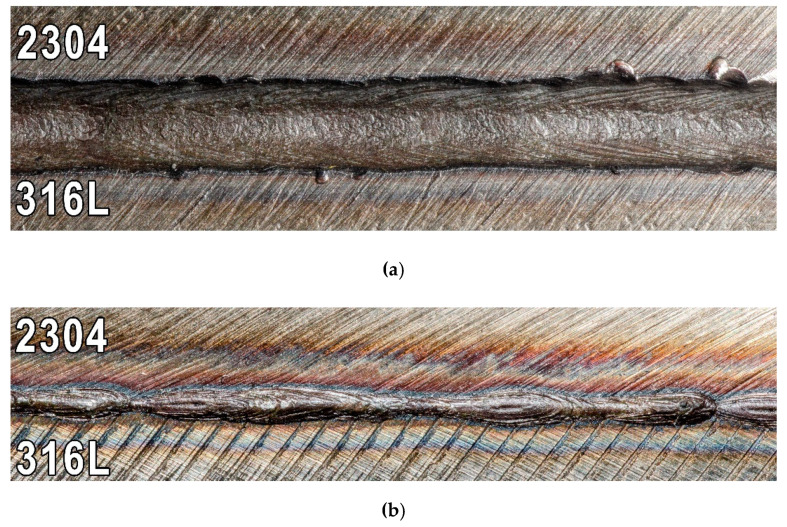
View of the welded joint: (**a**) face side and (**b**) root side.

To detect surface imperfections VT and PT were conducted. Positive results of those tests were found for all specimens. Figure 2 shows face and root side of one of the welded joints.

Figure 2a shows the view of the face of welded joint. Underfilling of the weld face can be seen, which in the case of laser welding of large thicknesses without consumable is expected. The welded joints met the assumed acceptance criterion of quality level B in accordance with the EN ISO 13919–1 standard. Therefore, on a basis of NDT results it was determined that the next part of the research, consisting of destructive tests, can be carried out. 

### 3.2. Destructive Tests

#### 3.2.1. Static Tensile Test 

**Figure 3:**
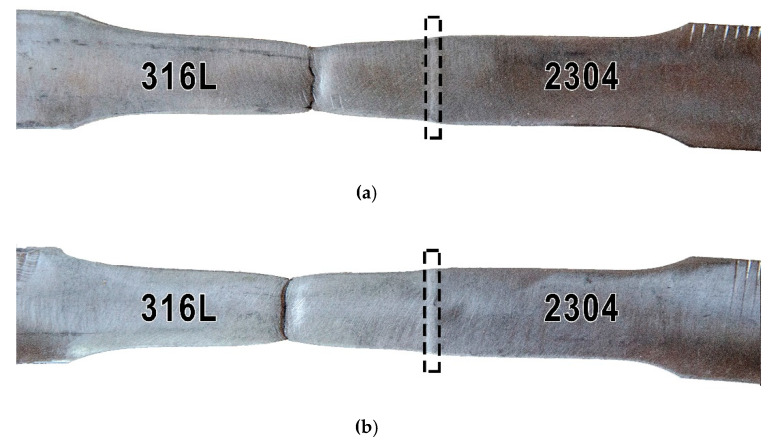
View of the specimens after a static tensile test: (**a**) specimen A and (**b**) specimen B. Welds marked by dashed contour lines.

Figure 3 shows the view of two specimens (signed A and B) which were subjected to transverse tensile tests. The results of the test are presented in Table 3. Minimum tensile strength required for the 316L austenitic stainless steel should be 530 MPa, and minimum tensile strength required for the 2304 duplex stainless steel should be 630 MPa (Table 2). For this test, the acceptance criterion was to exceed the minimum tensile strength of 316L austenitic stainless steel.

The obtained results meet the acceptance criterion and the obtained values are 10–15% higher compared to the requirements. The fracture was ductile and occurred in the 316L base material. It proves the correctness of welding parameters in terms of laser beam power, focus position and welding speed. The identified welding imperfections in the VT and PT tests did not affect the tensile strength. Tests showed that no structural changes reducing mechanical properties occurred under the influence of the laser welding thermal cycle. The absence of structural changes will be verified by metallographic examinations (Section 3.2.3).

#### 3.2.2. Bending Test

**Figure 4:**
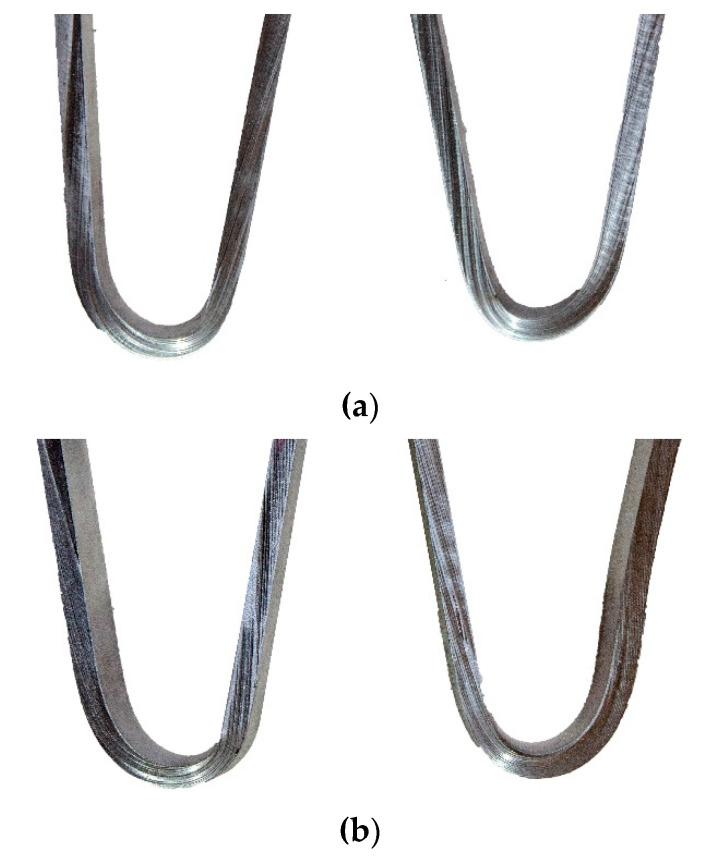
Specimens after bending tests: (**a**) bending from the face and (**b**) bending from the root.

The bending tests were carried out on four specimens—in two the tensiled side was the face of the weld and in two the tensiled side was the root of the weld. The aim of this test was to investigate the plastic properties of the welded joint. The bending tests are also carried out to reveal welding imperfections, e.g., incomplete fusion, lack of penetration, pores, and others. Specimens after the bending test are presented in Figure 4.

The bending test was executed until an angle of 180° was reached. The welds were subjected to significant plastic deformation and no surface cracks were visible on the root or face side, which indicates that the laser welded dissimilar joints exhibited good ductility and adequate bending strength. Such a test result proves very good plastic properties, but also a lack of welding imperfections in the tested specimens. As in the tensile test, underfill did not lead to cracks on the tensiled surfaces during the bending test. 

#### 3.2.3. Macro- and Microscopic Examinations

**Figure 5:**
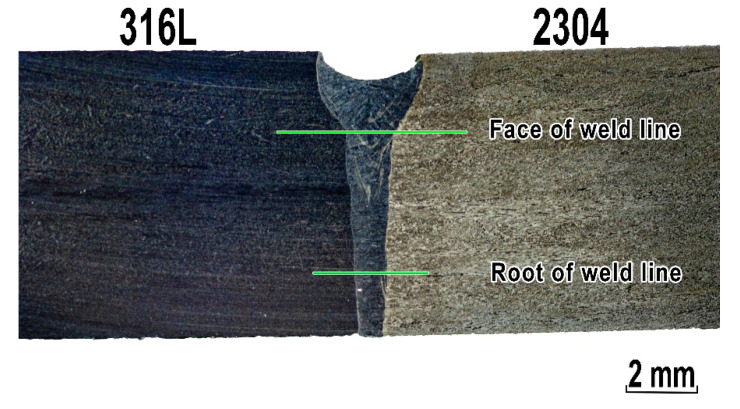
Cross-section of the 316L–2304 stainless steel welded joint. The arrangement of microhardness measurement points on the specimen is marked by lines.

Figure 5 presents the macrostructure of the welded joint observed on the cross-section of the weld axis. The joint has a regular symmetrical shape with visible underfilling of the weld face, without visible pores or excess penetration from the root side. Typical geometry of laser welded joint was observed. Keyhole laser welding forms a ‘chalice’ shaped weld bead profile. Examined welded joints were made without welding imperfections such as porosity and humping beads. Full penetration was achieved for investigated welded joints without changing beam focusing position.

**Figure 6:**
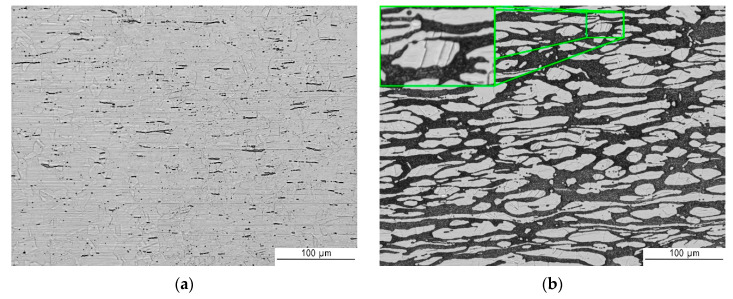
Metallographic structure (light microscope (LM)) of: (**a**) 316L austenitic stainless steel and (**b**) 2304 duplex stainless steel.

Figure 6 shows the structure of base materials: 316L austenitic stainless steel and 2304 lean duplex stainless steel. Figure 6a presents a microstructure of 316L including twins, slip bands, and δ ferrite (darker due to Beraha reagent etching). Austenite grains are quite fine, elongated in the direction of forming and between them big amount of darker δ ferrite precipitates. As demonstrated in Figure 6b base material of 2304 lean duplex steel is characterized by a dark continuous matrix of the ferrite phase (δ) and white island of austenitic (γ) phase with characteristic twins made visible through two-stage etching. Visible directionality of the structure is related to the rolling process. 

**Figure 7:**
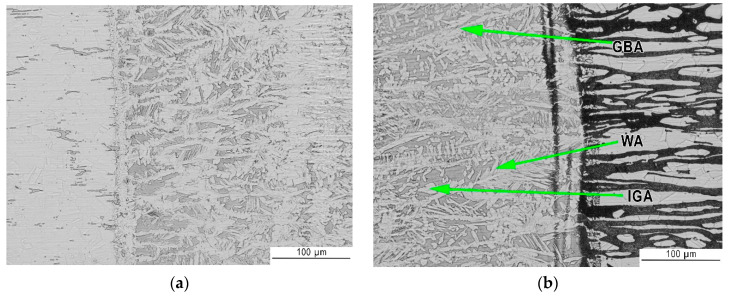
Metallographic structure (LM) of: (**a**) 316L austenitic stainless steel→HAZ→WM and (**b**) WM→HAZ→2304 duplex stainless steel.

**Figure 8:**
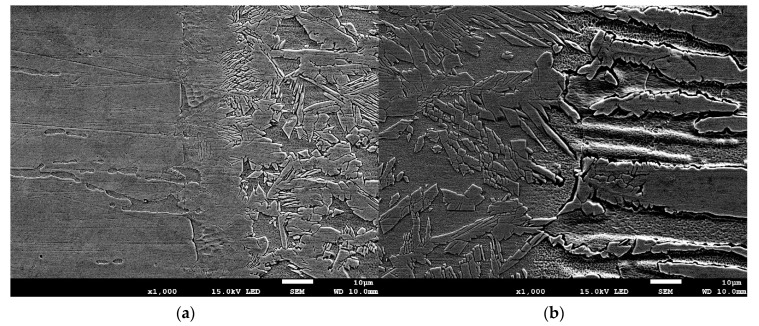
Metallographic structure (SEM) of: (**a**) 316L austenitic stainless steel→HAZ→WM and (**b**) WM→HAZ→2304 duplex stainless steel.

Figure 7 and Figure 8 show the transition areas from base material through the HAZ to the weld metal (WM). The austenitic structure of the weld consists of equiaxed coarse and fine dendrites. In addition, no eutectics or microcracks were found in the weld structure. During cooling the liquid metal in welding pool firstly solidify as ferrite. Further cooling cause partial transformation of ferrite to austenite. Austenite initially form as grain boundary allotriomorphic austenite (GBA), then as Widmanstätten side plates of austenite (WA) and finally as intragranular precipitates of austenite (IGA). In general, the first two types of austenite (GBA and WA) require less driving force than intragranular needle austenite, which means that the grain boundary and Widmanstätten austenite formed earlier at higher temperatures, whereas intragranular austenite particles precipitated on further cooling at a lower temperature [55,56]. GBA morphology resembles a coherent island arranged along the border of ferrite. WA morphology can be described as small needles in a ferrite matrix. While the IGA morphology is like square islands in a ferrite matrix.

As presented in Figure 7 heat affected zones were barely visible. HAZ of laser welded stainless steel joints is very narrow, in this case it was about 20 µm for 316L steel and 50 µm for 2304 steel as can be seen on Figure 8a and b, respectively. Both for lower (LM—Figure 7) and higher magnifications (SEM—Figure 8), a difference in the austenite-ferrite ratio in the weld close to HAZ is noticeable. As expected, from the 316L steel side there is a larger amount of austenite in the weld metal than from the 2304 steel side.

**Figure 9:**
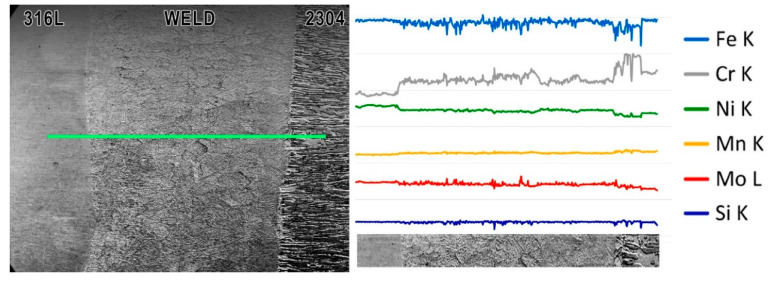
Results of EDS linear analysis through the welded joint from 316L austenitic stainless steel (beginning of the line) to 2304 duplex stainless steel (end of the line). The analysis was carried out along the line indicated in the SEM image on the left.

Linear EDS analysis was carried out through all welded joint: from 316L steel, first HAZ, weld metal, second HAZ ending at 2304 steel (Figure 9). The distribution of the analyzed elements along the measuring line is visible on individual curves. Level of the line does not show the content of a given element in the analyzed alloy, but only show the variability of its content along the measuring line. As could be predicted, the weld metal has a composition resulting from mixing of both materials—due to autogenous laser welding. The content of nickel which is stabilizing austenite element increases towards 316L steel. The heterogeneous composition in the weld metal can be the reason for the different austenite-ferrite ratio on both sides of the weld axis. Due to diffusion, differences in elements content occur in HAZ. The nickel content in HAZ next to 2304 steel decreases compared to the weld. The chromium content increases in the HAZ on the 2304 steel side. This increase may be due to a visible larger ferrite grains in this area and chromium is ferrite stabilizer. This can be seen in the photo below the graph (Figure 9), and is the effect of heat affecting the structure.

The low chromium content in the welding pool caused the formation of a structure with a higher content of austenite than is observed in duplex laser-welded joints with the same parameters and geometry [56]. As a result, the austenite to ferrite ratio is closer to 50:50. The obtained volume fraction of austenite may also be induced by the difference in the thermal conductivity coefficient of the austenitic steel in relation to the duplex steel, which resulted in an increase in the cooling time and allowed the transformation of ferrite into austenite [31].

Metallographic microscopic examination—SEM and EDS analysis—did not show the segregation of alloying elements between the ferritic and austenitic phases. This indicates that the areas depleted in Cr and Ni, which can cause degradation phenomena, e.g., corrosion, were not formed.

#### 3.2.4. Microhardness Measurements

**Figure 10:**
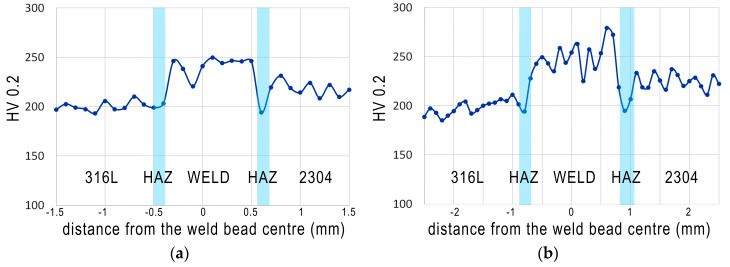
Microhardness (HV0.2) distribution across the welded joint: (**a**) 2 mm below the weld face and (**b**) 2 mm above the weld root.

Microhardness measurements were made in base materials, weld metal and HAZs in two lines as can be seen in Figure 5. Figure 10 shows the results of the measurements.

For 316L austenitic stainless steel microhardness value was in a range of 186–209 HV0.2, which corresponds well to the literature [38,50]. The microhardness fluctuations in the 316L steel are due to the presence of a large number of delta ferrite precipitates in the austenitic structure. In the HAZ (on 316L steel side) microhardness values are lower (average 196 HV0.2), which confirms that both heat input and cooling rate were appropriate for this process. The average HV0.2 value for weld metal is 254 HV0.2, with higher values on weld face than in the weld root. Differences in hardness values between the line passing through the face of the weld in relation to the line close to the root can be explained by differences in the amount of heat accumulated in each area. The average value of hardness for 2304 steel was 233 HV0.2—typical for this grade. However, its decrease in the HAZ on 2304 steel side (average value 197 HV0.2) was observed, which is a result of the structural changes shown in the microscopic studies. Due to the different morphology and austenite arrangement between weld metal and lean duplex, the microhardness of the autogenous weld metal was higher (about 20 HV0.2) than microhardness of the lean duplex steel [33]. The results are distinctive for dissimilar austenitic—duplex stainless steel welded joints—which was also demonstrated by other authors [33,52,54].

#### 3.2.5. Ferrite Content Measurements and Calculations

Points for ferrite measurement were chosen within the areas of 316L austenitic stainless steel and 2304 lean duplex stainless steel on the welded joint surface. Similarly, points were selected along the weld from the face and root of the weld. Table 4 shows the results of ferrite content measurements. 

The results of the delta ferrite measurements showed its correct, expected content in base materials. However, differences in the ferrite content between the face and the root of the weld were observed. Higher (by about 37%) ferrite content in the weld root is a consequence of different heat distribution between the border surfaces during and after the welding process. This distribution is also associated with various values of the thermal conductivity coefficient of base materials and the intensity of the laser heat source. Laser welding is characterized by high power density, which means that the energy distribution is constant over the entire depth (that’s why a cylindrical model of a heat source is usually used for numerical simulations).

Geometry of the weld is not constant throughout the thickness of the specimen, but it changes significantly in the area of the weld face (Figure 5). Forming a ‘chalice’ shaped weld bead is characteristic for the keyhole laser welding of thick materials. The change in the fusion line shape also indicates different heat distribution conditions in the welding area. This causes a change in the cooling rate of the joint on the upper and lower boundary surface (face/root). This phenomenon and different thermal properties can be explained by the increase in ferrite content in the weld root, as this leads to an increase in the cooling rate of the joint.

**Figure 11:**
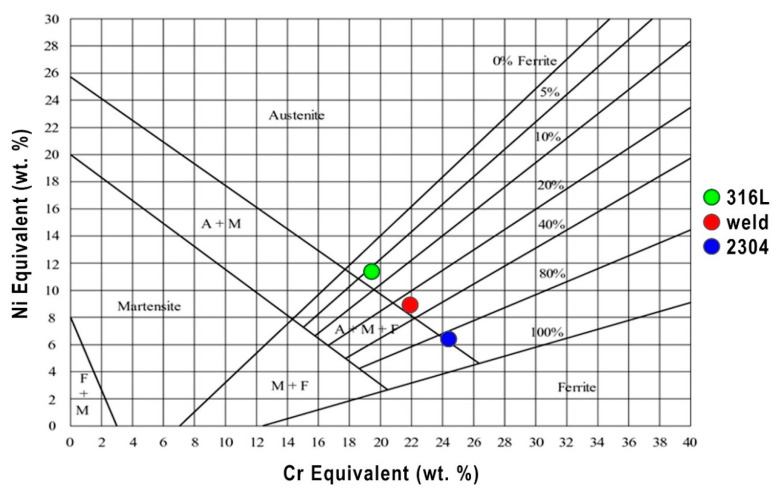
Schaeffler constitutional diagram.

The results of ferrite content measurements were compared with the results obtained from the Schaeffler diagram—a graphic method for assessing the weldability of high alloy steels and dissimilar joints. Schaeffler diagram is a good preliminary prediction method for weldability of steels subjected to fusion welding [54]. It was assumed, according to the welding conditions presented in Chapter 2, that a dilution rate was 50%, so the values of Creq and Nieq for the weld metal are 21.9% and 8.9% respectively, and this corresponds to about 30% of the amount of ferrite (Figure 11). Comparative analysis of the results of measurements by the magnetic method and the Schaeffler diagram confirm that Creq and Nieq in this form does not fully describe the ferrite forming tendency during laser welding. Obtained results of prediction of ferrite content were also confirmed using WRC-92 diagram.

**Figure 12:**
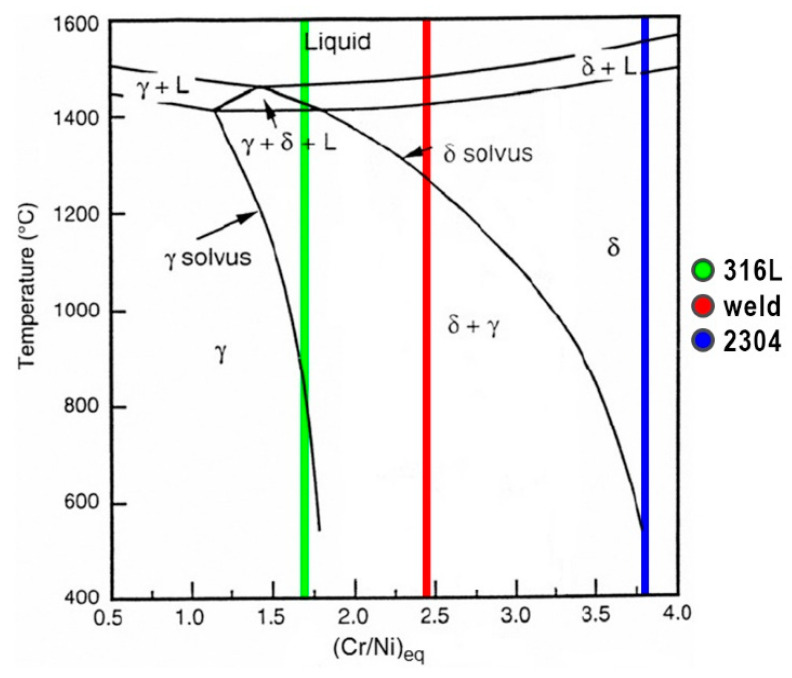
Fe-Ni-Cr pseudo-binary phase diagram.

Solidification mode of stainless steels can be predicted by the Fe–Ni–Cr pseudo-binary phase diagram shown in Figure 12 based on Creq/Nieq ratio [50]. As a result of welding of 316L steel without filler metal (Creq/Nieq = 1.71) the weld solidification mode is ferrite–austenite (FA). According to the Figure 12, Creq/Nieq ratio of 2304 steel equal to 3.80 during rapid cooling is enough to cause δ-ferrite solidification and then ferrite and austenite transformation (ferrite mode—F). Chemical composition of the weld metal, which is an alloy of 50% dilution rate, described by the value of Creq/Nieq = 2.46 also causes solidification in the F mode. The results of these analyzes are generally consistent with the results of metallographic examinations and ferrite content measurements.

## 4. Conclusions

Tests regarding dissimilar laser welding of butt joints made of 316L austenitic stainless steel and 2304 lean duplex stainless steel showed that the use of IPG YLS–6000 fiber laser with a maximum power of 6 kW allowed to obtain sound butt joints meeting the requirements of quality level B in accordance with EN ISO 13919–1 standard. During welding, the only imperfection detected by VT and PT was the underfilling of the face, while microscopic examinations did not show the presence of any other welding imperfections. Due to the very low (as for laser welding) welding speed, it was possible to achieve full penetration of 8 mm thick plates with one-sided course of the laser beam.

Obtained joints have good strength properties (higher than the minimum values required by the standard), which were confirmed in the static tensile tests (average R_m_ = 600 MPa). All specimens fractured in 316L austenitic stainless steel BM. The bending tests showed that plastic properties of base materials were not deteriorated and confirmed the absence of welding imperfections.

The microstructure of the dissimilar welded joint has a better austenite to ferrite ratio (closer to 50:50), compared to the microstructure observed on similar duplex steel welded joints [56]. This is caused by mixing of 316L steel and 2304 steel as well as by lower thermal conductivity of austenitic steel, which extends the cooling process and the time for austenite formation. Within the weld face, the weld width is greater, which affects a different amount of heat to be dissipated by the base material than in the case of weld root. The differences in thermal cycles, in these areas, cause changes in weld microhardness in the face and in the root of the weld. The microhardness of the autogenous weld metal (average 254 HV0.2) is higher than microhardness of the 2304 lean duplex stainless steel (average 233 HV0.2). The solidification microstructures of laser welds are generally consistent with the prediction from the Schaeffler diagram.

Based on the results of present study, autogenous fiber laser welding of 316L austenitic and 2304 lean duplex stainless steels without using a ceramic backing can be recommended as a suitable procedure for sound welded joints.

## Figures and Tables

**Table 1:** Chemical composition of base materials, wt.%.

Material	Value	C	Si	Mn	Ni	Cr	Mo	Cu	N	S	P	Creq	Nieq
316L	min	0.000	0.00	0.0	10.00	16.50	2.00	-	0.000	0.000	0.000		
	max	0.030	0.75	2.0	13.00	18.00	2.50	-	0.100	0.015	0.045		
	analysis	0.024	0.43	1.3	10.02	16.74	2.04	-	0.026	0.003	0.028	19.43	11.39
2304	min	0.000	0.0	0.0	3.5	22.0	0.10	0.10	0.05	0.000	0.000		
	max	0.030	1.0	2.0	5.5	24.0	0.60	0.60	0.20	0.015	0.035		
	analysis	0.027	0.4	1.4	4.9	23.4	0.39	0.32	0.14	0.001	0.029	24.39	6.41

**Table 2:** Mechanical and physical properties of base materials.

Material	Value	Rp0.2 (MPa)	Rm (MPa)	A50 (%)	λ (W/(m·K))
316L	min	220	530	40	16.2
	max		680		
2304	min	400	630	25	14.3
	max		800		

**Table 3:** Tensile test results of welded joints.

No Specimen	Cross-Section Area (mm^2^)	Maximum Loading Force (kN)	Tensile Strength, R_m_ (MPa)	Place of Fracture
1	170.7	104	609	Base material 316L
2	170.7	101	592	Base material 316L

**Table 4:** Experimental results of ferrite content.

Place of Measure	Ferrite Content (%)						Average Value	Standard Deviation
316L	0.23	0.24	0.23	0.26	0.25	0.24	0.24	0.01
2304	43.4	45.6	45.4	44.3	45.3	49.7	45.62	2.17
Weld face	13.9	12.6	13.0	14.9	11.3	12.0	12.95	1.30
Weld root	19.1	18.3	23.5	19.4	23.2	22.0	20.92	2.26

## References

[1] Quazi M.M., Ishak M., Fazal M.A., Arslan A., Rubaiee S., Aiman M.H., Qaban A., Yusof F., Sultan T., Ali M.M. (2020). A comprehensive assessment of laser welding of biomedical devices and implant materials: Recent research, development and applications. Crit. Rev. Solid. State.

[2] Kik T. (2020). Computational techniques in numerical simulations of arc and laser welding processes. Materials.

[3] Górka J. (2020). Assessment of the effect of laser welding on the properties and structure of TMCP steel butt joints. Materials.

[4] Lisiecki A. (2019). Study of optical properties of surface layers produced by laser surface melting and laser surface nitriding of titanium alloy. Materials.

[5] Janicki D., Górka J., Kwaśny W., Pakieła W., Matus K. (2020). Influence of solidification conditions on the microstructure of laser-surface-melted ductile cast iron. Materials.

[6] Sroka M., Jonda E., Pakieła W. (2020). Laser surface modification of aluminium alloy AlMg9 with B4C powder. Materials.

[7] Wang G., Wang J., Yin L., Hu H., Yao Z. (2020). Quantitative correlation between thermal cycling and the microstructures of X100 pipeline steel laser-welded joints. Materials.

[8] Lisiecki A., Kurc-Lisiecka A. (2018). Automated laser welding of AISI 304 stainless steel by disk laser. Arch. Metall. Mater..

[9] Tęczar P., Majkowska-Marzec B., Bartmański M. (2019). The influence of laser alloying of Ti13Nb13Zr on surface topography and properties. Adv. Mater. Sci..

[10] Herthoge M., De Pelsmaeker J., Boone M., De Baere I., Van Paepegem W., Van Vlierberghe S. (2020). Laser welding of carbon fiber filled polytetrafluoroethylene. J. Mater. Proc. Technol..

[11] Liao H., Zhu J., Chang S., Xue G., Zhu H., Chen B. (2020). Al_2_O_3_ loss prediction model of selective laser melting Al_2_O_3_–Al composite. Ceram. Int..

[12] Wen X., Jin G., Cui X., Feng X., Lu B., Cai Z., Zhao Y., Fang Y. (2019). Underwater wet laser cladding on 316L stainless steel: A protective material assisted method. Opt. Laser Technol..

[13] Fu Y., Guo N., Cheng Q., Zhang D., Feng J. (2020). In-situ formation of laser-cladded layer on Ti-6Al-4 V titanium alloy in underwater environment. Opt. Lasers Eng..

[14] Kik T., Górka J. (2019). Numerical simulations of laser and hybrid S700MC T-joint welding. Materials.

[15] Wang H., Liu X., Liu L. (2020). Research on laser-TIG hybrid welding of 6061-T6 aluminum alloys joint and post heat treatment. Metals.

[16] Yazdian N., Mohammadpour M., Razavi R., Kovacevic R. (2018). Hybrid laser/arc welding of 304L stainless steel tubes, part 2–Effect of filler wires on microstructure and corrosion behavior. Int. J. Press. Vessel. Pip..

[17] Mohammed G.R., Ishak M., Aqida S.N., Abdulhadi H.A. (2017). Effects of heat input on microstructure, corrosion and mechanical characteristics of welded austenitic and duplex stainless steels: A review. Metals.

[18] Kuryntsev S.V., Gilmutdinov A.K. (2015). Welding of stainless steel using defocused laser beam. J. Constr. Steel Res..

[19] Verma J., Taiwade R.V. (2017). Effect of welding processes and conditions on the microstructure, mechanical properties and corrosion resistance of duplex stainless steel weldments—A review. J. Manuf. Process..

[20] Prijanovič U., Prijanovič Tonkovič M., Trdan U., Pleterski M., Jezeršek M., Klobčar D. (2020). Remote fibre laser welding of advanced high strength martensitic steel. Metals.

[21] Pańcikiewicz K., Radomski W. (2020). Lack of tightness analysis of concealed welded radiators. Eng. Fail. Anal..

[22] Skowrońska B., Chmielewski T., Pachla W., Kulczyk M., Skiba J., Presz W. (2019). Friction weldability of UFG 316L stainless steel. Arch. Metall. Mater..

[23] Krella A.K., Krupa A. (2018). Effect of cavitation intensity on degradation of X6CrNiTi18-10 stainless steel. Wear.

[24] Kuryntsev S.V. (2019). Effect of heat treatment on the phase composition and corrosion resistance of 321 SS welded joints produced by a defocused laser beam. Materials.

[25] Świerczyńska A., Fydrych D., Landowski M., Rogalski G., Łabanowski J. (2020). Hydrogen embrittlement of X2CRNiMoCuN25-6-2 super duplex stainless steel welded joints under cathodic protection. Constr. Build. Mater..

[26] Varbai B., Májlinger K. (2019). Optimal etching sequence for austenite to ferrite ratio evaluation of two lean duplex stainless steel weldments. Measurement.

[27] Ouali N., Khenfer K., Belkessa B., Fajoui J., Cheniti B., Idir B., Branchu S. (2019). Effect of heat input on microstructure, residual stress, and corrosion resistance of UNS 32101 lean duplex stainless steel weld joints. J. Mater. Eng. Perform..

[28] Verma J., Taiwade R.V. (2016). Dissimilar welding behavior of 22% Cr series stainless steel with 316L and its corrosion resistance in modified aggressive environment. J. Manuf. Proc..

[29] Świerczyńska A., Łabanowski J., Michalska J., Fydrych D. (2017). Corrosion behavior of hydrogen charged super duplex stainless steel welded joints. Mater. Corros..

[30] Varbai B., Májlinger K. (2019). Physical and theoretical modeling of the nitrogen content of duplex stainless steel weld metal: Shielding gas composition and heat input effects. Metals.

[31] Yang Y., Guo Y., Liu Y., Li J., Jiang Y. (2019). The microstructure and pitting resistance of 2002 lean duplex stainless steel after the simulated welding thermal cycle process. Materials.

[32] Łabanowski J., Fydrych D., Rogalski G., Samson K. (2012). Underwater welding of duplex stainless steel. Solid State Phenom..

[33] Silva Leite C.G., da Cruz Junior E.J., Lago M., Zambon A., Calliari I., Ventrella V.A. (2019). Nd: YAG pulsed laser dissimilar welding of UNS S32750 duplex with 316L austenitic stainless steel. Materials.

[34] Fydrych D., Łabanowski J., Tomków J., Rogalski G. (2015). Cold cracking of underwater wet welded S355G10+N high strength steel. Adv. Mater. Sci..

[35] Rogalski G., Świerczyńska A., Landowski M., Fydrych D. (2020). Mechanical and microstructural characterization of TIG welded dissimilar joints between 304L austenitic stainless steel and Incoloy 800HT nickel alloy. Metals.

[36] Tomków J., Fydrych D., Rogalski G. (2020). Dissimilar underwater wet welding of HSLA steels. Int. J. Adv. Manuf. Tech..

[37] Wang W., Hu Y., Zhang M., Zhao H. (2020). Microstructure and mechanical properties of dissimilar friction stir welds in austenitic-duplex stainless steels. Mater. Sci. Eng. A.

[38] Ramkumar K.D., Singh A., Raghuvanshi S., Bajpai A., Solanki T., Arivarasu M., Arivazhagan N., Narayanan S. (2015). Metallurgical and mechanical characterization of dissimilar welds of austenitic stainless steel and super-duplex stainless steel–a comparative study. J. Manuf. Process..

[39] Yıldızlı K. (2015). Investigation on the microstructure and toughness properties of austenitic and duplex stainless steels weldments under cryogenic conditions. Mater. Des..

[40] Başyiğit A.B., Kurt A. (2017). Investigation of the weld properties of dissimilar S32205 duplex stainless steel with AISI 304 steel joints produced by arc stud welding. Metals.

[41] Abdollahi A., Shamanian M., Golozar M.A. (2018). Comparison of pulsed and continuous current gas tungsten arc welding in dissimilar welding between UNS S32750 and AISI 321 in optimized condition. Int. J. Adv. Manuf. Tech..

[42] Theodoro M.C., Pereira V.F., Mei P.R., Ramirez A.J. (2015). Dissimilar friction stir welding between UNS S31603 austenitic stainless steel and UNS S32750 superduplex stainless steel. Metall. Mater. Trans. B.

[43] Neissi R., Shamanian M., Hajihashemi M. (2016). The effect of constant and pulsed current gas tungsten arc welding on joint properties of 2205 duplex stainless steel to 316L austenitic stainless steel. J. Mater. Eng. Perform..

[44] Szala M., Łukasik D. (2018). Pitting corrosion of the resistance welding joints of stainless steel ventilation grille operated in swimming pool environment. Int. J. Corros..

[45] Vicente T.A., Oliveira L.A., Correa E.O., Barbosa R.P., Macanhan V.B.P., Alcântara N.G. (2018). Stress corrosion cracking behaviour of dissimilar welding of AISI 310S austenitic stainless steel to 2304 duplex stainless steel. Metals.

[46] Rahmani M., Eghlimi A., Shamanian M. (2014). Evaluation of microstructure and mechanical properties in dissimilar austenitic/super duplex stainless steel joint. J. Mater. Eng. Perform..

[47] Moteshakker A., Danaee I. (2016). Microstructure and corrosion resistance of dissimilar weld-joints between duplex stainless steel 2205 and austenitic stainless steel 316L. J. Mater. Sci. Technol..

[48] Moteshakker A., Danaee I., Moeinifar S., Ashrafi A. (2016). Hardness and tensile properties of dissimilar welds joints between SAF 2205 and AISI 316L. Sci. Technol. Weld. Join..

[49] Taheri A., Beidokhti B., Boroujeny B.S., Valizadeh A. (2020). Characterizations of dissimilar S32205/316L welds using austenitic, super-austenitic and super-duplex filler metals. Int. J. Min. Metall. Mater..

[50] Fei Z., Pan Z., Cuiuri D., Li H., Van Duin S., Yu Z. (2019). Microstructural characterization and mechanical properties of K-TIG welded SAF2205/AISI316L dissimilar joint. J. Manuf. Process..

[51] Ramkumar K.D., Bajpai A., Raghuvanshi S., Singh A., Chandrasekhar A., Arivarasu M., Arivazhagan N. (2015). Investigations on structure–property relationships of activated flux TIG weldments of super-duplex/austenitic stainless steels. Mater. Sci. Eng. A.

[52] Ridha Mohammed G., Ishak M., Ahmad S.N.A.S., Abdulhadi H.A. (2017). Fiber laser welding of dissimilar 2205/304 stainless steel plates. Metals.

[53] Chun E.J., Lee J.H., Kang N. (2019). Unmixing behaviour in dissimilar laser welds for duplex and austenitic stainless steels. Sci. Technol. Weld. Join..

[54] Saravanan S., Raghukandan K., Sivagurumanikandan N. (2017). Studies on metallurgical and mechanical properties of laser welded dissimilar grade steels. J. Braz. Soc. Mech. Sci. Eng..

[55] Cao F., Zhang Y., Shen Y., Jin Y., Li J., Hou W. (2020). Effects of beam offset on the macro defects, microstructure and mechanical behaviors in dissimilar laser beam welds of SDSS2507 and Q235. J. Manuf. Process..

[56] Landowski M. (2019). Influence of parameters of laser beam welding on structure of 2205 duplex stainless steel. Adv. Mater. Sci..

